# Incidence of burns infections: interest of a protocol for toilet and dressing (observational study before / after)

**DOI:** 10.1186/2197-425X-3-S1-A1010

**Published:** 2015-10-01

**Authors:** S Wiramus, C Sartor, P Ainaud, V Bernini, J Albanese

**Affiliations:** APHM CHU Conception, Burn Center, Marseille, France; APHM CHU Conception, Infectious Disease, Marseille, France

## Introduction

Infections are a major cause of morbidity and mortality in intensive care. In burned patients, the risk of infection is increased by disruption of skin barrier. Colonization and skin infections delay the healing, compromise skin grafts and increase hospital stay. With a high rate of positive microbiological sampling over several months, we decided to evaluate the incidence of skin infections in the burn center and design any corrective measures.

## Objectives

Primary objective: to evaluate the effectiveness of a cleaning protocol for toilets and dressing on the incidence of skin colonization and infection. Secondary objectives: time between the burn and the positive microbiological sampling, bacterial epidemiology.

## Methods

Single-center retrospective study. Before the study, daily toilet, preoperative and operative toilets were not protocolized : either chlorhexidine or povidone iodine or both. With the assistance of the hospital comity about nosocomial infections, we studied the clinical practice of the unit and compared them to the recommendations, taking into account the specificities of a burned patient. This study is based on medical records of patients admitted more than 24 hours with at least one skin bacteriological sample (biopsy, swab). The "skin infection" was defined on clinical appearance and skin biopsy ≥ 105 CFU; we talked about colonization if < 105 CFU. Data were analyzed using the statistical software "R". A descriptive analysis was performed and quantitative data are presented as median and interquartile range whereas qualitative data as absolute count and percentage. To compare variables between the groups we used a Chi2 test. We choose a p-value of less than 0.05 to consider a significant difference.

## Results

**Figure 1:**
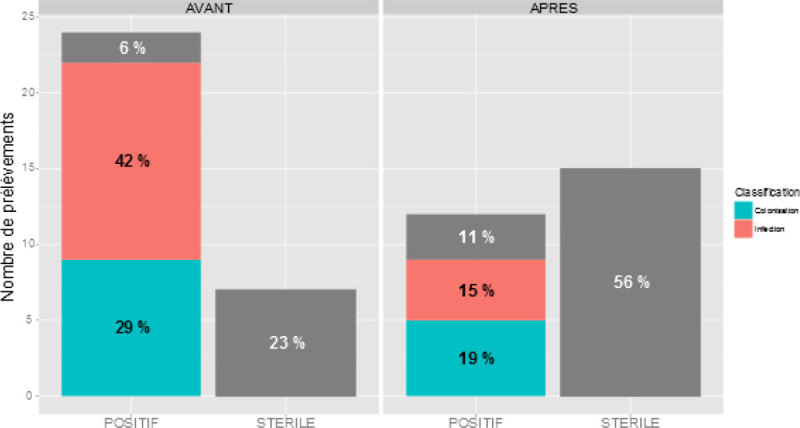
Figure 1

This skin cleaning protocol of burns (0.05% chlorhexidine or povidone iodine before surgery) has reduced the incidence of skin infections from 42 to 15% (p = 0.03) and colonization skin from 29 to 19% (p = 0.03) (Figure 1). The time between the burn and positive skin sample was similar between the two groups (14 vs 13 days, ns). No side effects were noted for the use of antiseptics.

## Conclusions

With this study, we allowed to reduce significantly the rate of positive skin samples in our patients (p = 0.015). Recurrent evaluation of clinical practice allows to adjust the protocols and improve care. Regular epidemiological studies also let to identify peaks of infection related to potential inadequate clinical practice.

